# Exceptionally high cumulative percentage of NUMTs originating from linear mitochondrial DNA molecules in the *Hydra magnipapillata* genome

**DOI:** 10.1186/1471-2164-14-447

**Published:** 2013-07-04

**Authors:** Shen Song, Feng Jiang, Jianbo Yuan, Wei Guo, Yongwang Miao

**Affiliations:** 1 Faculty of Animal Science and Technology, Yunnan Agricultural University, Kunming, Yunnan 650201, China; 2 Institute of Zoology, Chinese Academy of Sciences, Beijing 100101, China; 3 Beijing Institutes of Life Science, Chinese Academy of Sciences, Beijing 100101, China; 4 Institute of Oceanology, Chinese Academy of Sciences, Qingdao 266071, China

**Keywords:** Cnidaria, Mitochondrial DNA, NUMT, Linear mitochondrial genome

## Abstract

**Background:**

In contrast to most animal genomes, mitochondrial genomes in species belonging to the phylum Cnidaria show distinct variations in genome structure, including the mtDNA structure (linear or circular) and the presence or absence of introns in protein-coding genes. Therefore, the analysis of nuclear insertions of mitochondrial sequences (NUMTs) in cnidarians allows us to compare the NUMT content in animals with different mitochondrial genome structures.

**Results:**

NUMT identification in the *Hydra magnipapillata*, *Nematostella vectensis* and *Acropora digitifera* genomes showed that the NUMT density in the *H. magnipapillata* genome clearly exceeds that in other two cnidarians with circular mitochondrial genomes. We found that *H. magnipapillata* is an exceptional ancestral metazoan with a high NUMT cumulative percentage but a large genome, and its mitochondrial genome linearisation might be responsible for the NUMT enrichment. We also detected the co-transposition of exonic and intronic fragments within NUMTs in *N. vectensis* and provided direct evidence that mitochondrial sequences can be transposed into the nuclear genome through DNA-mediated fragment transfer. In addition, NUMT expression analyses showed that NUMTs are co-expressed with adjacent protein-coding genes, suggesting the relevance of their biological function.

**Conclusions:**

Taken together, our results provide valuable information for understanding the impact of mitochondrial genome structure on the interaction of mitochondrial molecules and nuclear genomes.

## Background

In eukaryotes, mitochondrial DNA sequences are frequently transferred into the nuclear genome, generating nuclear mitochondrial DNA sequences (NUMTs) [1]. Du Buy and Riley identified NUMTs in a wide range of species, including plants, yeast, alveolates, nematodes, insects, and vertebrates [2-5]. Although NUMTs are present in numerous species, the numbers of these sequences widely vary. Some species (*Monodelphis domestica*) possess up to 2.04 Mbp of NUMTs, whereas no NUMTs have been detected in other species (e.g., *Anopheles gambiae*, *Branchiostoma floridae*, *Danio rerio*, *Ciona savignyi*) [4,5]. To date, the highest cumulative percentage of NUMTs is 0.0861% in the *Apis mellifera* genome [5,6]. NUMTs accumulate in the genomes over a continuous evolutionary process [7-11]. In general, each NUMT originates in one of two ways: the independent insertion of DNA from mitochondrial DNA into nuclear chromosomes and duplication after the insertion into nuclear DNA [9,12-15]. Previous studies in human and honeybee genomes have suggested that only one-third of NUMTs were integrated as independent mitochondrial sequences, whereas the remaining two-thirds of NUMTs arose from duplications after insertion into the nuclear genome [6,8]. Transposable elements or short-dispersed repeats have been associated with the on-going integration of mtDNA sequences into the nuclear genome and their subsequent duplication [16-18].

A large number of NUMTs are considered “dead on arrival” because these sequences are non-functional pseudo-genes, as evidenced through the presence of stop codons, frameshifts, and/or indels in their coding sequences [19,20]. However, evidence of functional NUMTs has been observed in a few species. For instance, five NUMTs within gene-coding regions have been identified in expressed sequence tags (ESTs) in the honeybee genome [9]. In addition, Ricchetti and coworkers identified 22 out of 28 human-specific NUMTs inserted in known or predicted introns, 1 NUMT in an exon, 1 NUMT in a promoter region, and 4 NUMTs in intergenic regions. These authors also suggested that NUMTs would preferentially integrate into coding or regulatory sequences and cause insertions associated with human diseases and those induced through environmental insults [21].

Cnidaria is one of the earliest branches in the animal tree of life, as evidenced through fossil records dating approximately 600 million years ago [22]. The mtDNA in the phylum Cnidaria represents a “hot spot” of mitochondrial genomic diversity in animals due to variation in both the gene content and mtDNA genome architecture [23]. The cnidarian mitochondrial genomes have two unique characteristics. Among the four traditionally recognised cnidarian classes, species in the Scyphozoan, Cubozoan, and Hydrozoan classes display linear mitochondrial genomes, while those in the Anthozoa class exhibit circular mitochondrial genomes [24,25]. The *H. magnipapillata* mitochondrial genome consists of two separate molecules: mitochondrial chromosome 1 (mt-Chr 1) and mitochondrial chromosome 2 (mt-Chr 2) [26]. Despite its fragmentary molecules, the length of the *H. magnipapillata* mitochondrial genome is 15,880 bp (8,194 bp and 7,686 bp, respectively), showing a typical metazoan mitochondrial genome size [23]. Identical inverted terminal repeats (ITR) occur on both *H. magnipapillata* mitochondrial chromosomes, similar to those in the *Aurelia aurita* and *H. oligactis* linear mitochondrial genome, and both mitochondrial chromosomes possess identical oriented sequences at the 5’ and 3’ ends (5’ and 3’ IOS) adjacent to the ITR [27,28]. Another unique characteristic of cnidarian mitochondrial genomes is that introns have been observed in several classes [29,30]. The *N. vectensis* and *A. digitifera* mitochondrial genome comprises molecular DNA containing a group I intron in the *ND5* gene. However, the genes identified in group I are different [30,31].

Our knowledge of mitochondrial DNA integrated into animal nuclear genomes is primarily limited to animals with circular mitochondrial genomes without introns. NUMT studies are not available in animals with linear mitochondrial genomes or those with intron-containing mitochondria. Therefore, it remains to be determined whether NUMT transposition differences exist between circular and linear mtDNAs [4,5]. The completion of the mitochondrial genome sequence and recent availability of the genome draft sequences of *Hydra magnipapillata*, *Nematostella vectensis*, and *Acropora digitifera* have led to the description of NUMT features in the nuclear genomes of these three cnidarian genomes [26,31-34].

Whole-genome shotgun sequencing was also used to sequence these three cnidarian genomes. Compared with the cost-effective data obtained from Illumina sequencing, the longer sequencing reads from Sanger sequencing or 454 pyro-sequencing were produced to assemble these genomes. Therefore, instead of the popular sequencing-by-hybridisation algorithm based on the *k*-mer content, overlap-layout-consensus and greedy algorithms were adopted for the assembly of the three cnidarian genomes. The advantage of genome assembly from long sequencing reads is that it provides a more accurate estimation of the NUMT content compared with genome assembly from short sequencing reads [32-34]. A description of NUMT features might provide information concerning the characteristics of mitochondrial pseudo-genes in these three species from the earliest branches in animal evolution, and it might also offer a comparison between NUMTs originating from circular mtDNA molecules and those originating from linear mtDNA molecules. In addition, the present study provides a detailed investigation on the genome-wide identification of NUMTs in three cnidarian species with distinct mitochondrial structures and explores the NUMT landscape in a species with linear mtDNA molecules.

## Results

### Exceptionally high frequency of NUMT insertions in the *H. magnipapillata* genome

**Figure 1:**
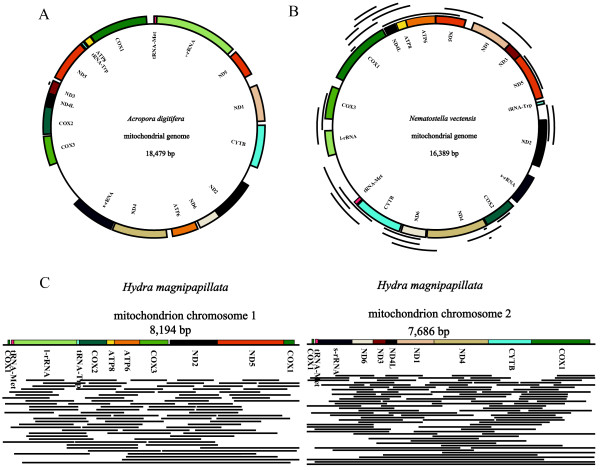
**NUMT distribution in three cnidarian mitochondrial genomes. (A)** NUMT distribution in the *A. digitifera* mitochondrial genome. The mtDNA genome contains the genes for 13 energy pathway proteins, two rDNAs and two tRNAs. A single intron occurs in the *ND5* gene. Note that the intron occurs within the *ND5* gene and contains the only copies of the *tRNA-Trp*, *ATP8*, *COX1*, *tRNA-Met*, and *l-rRNA* genes. The black line represents the only NUMT in the *A. digitifera* genome. **(B)** NUMT distribution in the *N. vectensis* mitochondrial genome. A single intron occurs in the *ND5* gene. Note that the intron occurs within the ND5 gene and contains the only copies of the *ND1* and *ND3* genes. Each black line represents a NUMT insertion in the reference genome. **(C)** NUMT distribution in the *H. magnipapillata* mitochondrial genome. For clarity, the NUMTs whose length is longer than 500 bp are indicated with black lines.

**Figure 2:**
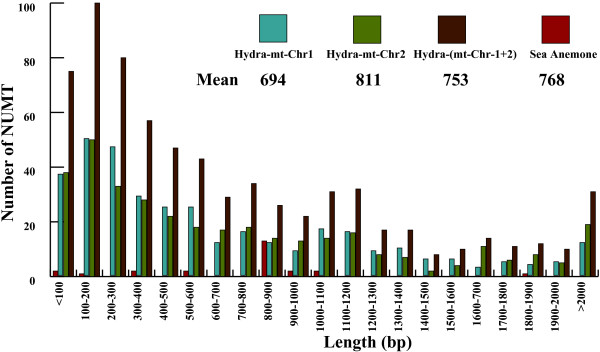
NUMT lengths distribution.

We determined the homology relationships between the three cnidarian nuclear genomes and their corresponding mitochondrial genomes using BLASTN searches. A total of 704, 24 and 1 NUMTs were inferred in *Hydra magnipapillata*, *Nematostella vectensis*, and *Acropora digitifera*, respectively (Figure 1, Additional file 1: Table S1, Table S2, Additional file 2: Table S3, and Additional file 3: Table S4). The length distribution of the BLASTN hits was 51 to 7,684 bp (mean: 753 bp) for *H. magnipapillata*, 76 to 1,836 bp (mean: 768 bp) for *N. vectensis*, and 86 bp for *A. digitifera* (Figure 2). No obvious differences were observed in the length of NUMTs between *H. magnipapillata* and *N. vectensis* (mean lengths: 753 bp and 768 bp, respectively; Student’s t test: *P* = 0.8536), while their mean lengths were much longer than those observed in many other metazoans (e.g., *Amphimedon queenslandica*: 222 bp; *Drosophila sechellia*: 240 bp; *Homo sapiens*: 309 bp; *Nasonia vitripenni*: 565 bp) [5,35]. The largest proportion of NUMTs was between 100 and 200 bp in *H. magnipapillata* and between 800 and 900 bp in *N. vectensis* (Figure 2). The sequence similarity detected using BLASTN between NUMTs and homologous mtDNA sequences was 79.34 to 100%, 92.11 to 100%, and 95.35% in *H. magnipapillata*, *N. vectensis*, and *A. digitifera*, respectively. The mtDNA fraction covered by NUMTs was lower in *N. vectensis* (72.68%) and *A. digitifera* (0.47%) compared with *H. magnipapillata* (100%) (Table 1). NUMTs accounted for approximately 0.0470% (529,934 bp) of the *H. magnipapillata* genome (Table 1 and Figure 1C), which was much higher than the 0.0052% in *N. vectensis* (Table 1 and Figure 1B) and 0.00002% (86 bp) in *A. digitifera* (Table 1 and Figure 1A). The cumulative percentage was used as a measure of NUMT content in the genomes examined in this study, suggesting that the estimation of the NUMT content was not affected by differences in genome assembly fragmentation (Additional file 4: Table S5). Thus, although *H. magnipapillata*, *N. vectensis*, and *A. digitifera* belong to the same phylum, the NUMT compositions of the genomes of these three species are quite different. The number of NUMTs in the *H. magnipapillata* genome clearly exceeds those of the *N. vectensis* and *A. digitifera* genomes.

**Table 1:** Sizes of mtDNA, nuclear genomes, and NUMTs detected through a BLASTN search

**Species**	**mtDNA**		**Nuclear genome**		**NUMTs**	
	**Total size (bp)**	**Transferred (%)**	**Total (Mbp)**	**GC (%)**	**bp**	**(%)**
*H. magnipapillata*	8,194 + 7,686	100	1,101	27.6	529,934	0.0470
*N. vectensis*	16,389	72.68	357	41.9	18,440	0.0052
*A. digitifera*	18,479	0.47	420	39	86	0.00002

Note: “Transferred” mtDNA represents the fraction of mtDNA that generated the NUMTs. All nuclear sequences homologous to mtDNA are included in the “NUMTs (bps)” column. The values in the “NUMTs (%)” column represent the ratio of NUMTs to the total size of the nuclear genome.

NUMTs have been observed in several metazoans, but they are rare or even absent in the oldest branch of animal evolution [5,35]. Unexpectedly, we detected NUMT proliferation in the *H. magnipapillata* genome. BLASTN searches within the *H. magnipapillata* genome assembly using two mtDNA molecules, mt-Chr 1 and mt-Chr 2 as queries yielded 353 and 351 NUMT sequences, respectively (Additional file 1: Table S1 and Table S2). As shown in Figure 1C, these NUMTs covered almost the entire *H. magnipapillata* mitochondrial genome. The similarities between NUMTs and their corresponding mitochondrial counterparts varied from 79.34 to 100% in mt-Chr 1 and 79.35 to 100% in mt-Chr 2. NUMTs from mt-Chr 1 and mt-Chr 2 showed extensive differences in length, ranging from 51 to 7,684 bp and 51 to 7,421 bp, respectively. The mean lengths of the mt-Chr 2-specific NUMTs were longer than those of the mt-Chr 1-specific NUMTs (mt-Chr 1-specific mean = 694 bp and mt-Chr 2-specific mean = 811 bp; Student’s t test: *P* = 0.0760; Figure 2). The median number of NUMT length in mt-Chr 1-NUMT, mt-Chr 2-NUMT and *N. vectensis*-NUMT was 464 bp (its variance: 572,384), 519 bp (its variance: 956,478), and 861 bp (its variance: 142,365), respectively. In the *H. magnipapillata* genome, 529,934 bp (0.0470%) comprised sequences corresponding to a mitochondrial origin (Table 1), which was approximately 33.37 times longer than the entire original mitochondrial genome. We did not detect any insertion preference for certain mitochondrial genes in the *H. magnipapillata* genome. Approximately equal amounts of NUMTs in both mitochondrial genomes indicated that there was no obvious bias in the transferred region of both mitochondrial genomes.

### Characterisation of NUMTs identified in the three cnidarian genomes

In the three cnidarian genomes, mitochondrial protein-coding genes were transferred into the nuclear genome at different frequencies. Of the 17 mitochondrial genes with NUMT insertions, 17 counterparts were identified in the *H. magnipapillata* genome, 15 counterparts were identified in the *N. vectensis* genome, and only 1 counterpart was identified in the *A. digitifera* genome. Mitochondrial genes with relatively complete structures were observed in both the *H. magnipapillata* and *N. vectensis* genomes.

Protein-coding genes, ribosomal genes, and tRNA genes were identified in *H. magnipapillata* NUMTs, showing varying insertion frequencies. In total, we identified 1,133 mitochondrial gene fragment counterparts in *H. magnipapillata*, including 257 relatively complete mitochondrial genes located in the 138 NUMTs. Among these, 13 mitochondrial protein-coding genes, 3 tRNAs, and 2 rRNA genes were detected. In contrast, of the 7 NUMTs inferred from the *N. vectensis* genome, only 9 relatively complete mitochondrial genes were detected, including 2 copies of *tRNA-Met* genes and 7 mitochondrial protein-coding genes. Unlike those of *H. magnipapillata* and *N. vectensis*, the NUMTs of *A. digitifera* lacked complete mitochondrial genes. The only NUMT identified in the *A. digitifera* genome was homologous to the *ND3* gene fragment. The *H. magnipapillata* mitochondrial genes were present in multiple copies in the nuclear genome, and the copy numbers of these genes ranged from 19 to 46. Most NUMTs detected in *H. magnipapillata* were non-functional pseudo-genes, as evidenced by the mutations and short insertions in the coding regions of these genes. However, few protein-coding genes in NUMTs could be translated using mitochondrial and universal codons (e.g., *ND4 L*, Additional file 5: Figure S1A), and several NUMT tRNAs could be folded into perfect structures (e.g., tRNA-Trp, Additional file 5: Figure S1B).

### NUMT duplications in the *H. magnipapillata* genome

As in other animals (e.g., human, honeybee), NUMT duplications have occurred in the *H. magnipapillata* genome [8,9]. Of all NUMTs in *H. magnipapillata* (n = 704), 39 mtDNA insertions were duplicated at least once in the nuclear genome. Of these 39 cases, 34 insertions were duplicated twice, 4 insertions were duplicated 3 times, and 1 insertion was duplicated 6 times (Additional file 6: Table S6). The duplicated NUMTs ranged in size from 53 to 2,000 bp, with mtDNA sequence identity ranging from 79.34 to 100%. However, NUMT duplication events were absent in the *N. vectensis* and *A. digitifera* genomes.

We identified at least 46 NUMTs that originated from neighbouring mtDNA regions but were located away from each other in the *H. magnipapillata* nuclear genome. We compared the sequences between pairs of NUMTs manually or using the RepeatMasker program. These sequences were classified into the following two groups: (1) low-complexity regions containing AT-rich or repetitive sequences; and (2) sequences containing transposable elements (Additional file 7: Figure S2).

### Insertion of NUMTs in nuclear genes

NUMTs in Cnidaria are located in non-genic or low-gene-density regions, consistent with most bilaterians [19,36]. We inferred the presence of 23, 1, and 0 intronic NUMTs in *H. magnipapillata*, *N. vectensis*, and *A. digitifera*, respectively. The genomic locations of NUMTs further revealed that most *H. magnipapillata* NUMTs (94.46%, 665 of 704) were located in non-genic regions (74.74%, 526 of 665) or low-gene-density regions (19.7%, 139 of 665). For *N. vectensis*, 87.5% (21 of 24) of the NUMTs were inserted in non-genic regions (52.38%, 11 of 24) or low-gene-density regions (47.62%, 10 of 24), while 12.5% (3 out of 24) of the NUMTs were located in regions of high gene density. However, we only detected one NUMT located in a non-genic region in *A. digitifera*.

We identified a fraction of NUMTs (3.25%, 23 of 704) in *H. magnipapillata* located in introns, similar to 1 NUMT from scaffold 1 of *N. vectensis* (Table 2). In *H. magnipapillata*, genes typically contained a single NUMT. For example, the *s-rRNA* gene, *ND4*, *ND1*, *COX1*, and *COX2* fragments were transposed into introns of *protein kinase C, delta*, *neuroendocrine convertase 1*, *sox10*, and *cytochrome b5 reductase 4*, respectively. Genes containing more than 1 NUMT were identified in 3 cases (Table 2). The only intron of the *mitoferrin-1 gene* contained 8 NUMTs, intron 3 of the *MAD homolog 4-interacting transcription coactivator 1* gene contained 4 NUMTs, and intron 2 of LOC100199452 contained 2 NUMTs. All NUMTs within a gene were in the same orientation (Additional file 8: Figure S3). Twelve mitochondrial genes had intronic NUMT counterparts. *ψCOX1* was identified in 8 different intronic NUMTs, the *s-rRNA* gene was detected in 5 intronic NUMTs, and all other genes in intronic NUMTs were present at a low frequency. No observed NUMT insertions overlapped with exons.

**Table 2:** NUMTs in the predicted nuclear genes

**Species**	**Nuclear host gene**	**No. of NUMT**	**Size (bp)**	**Mitochondrial origin**	**Host gene annotation**
*H. magnipapillata*	LOC100203425	1	914	COX2 + ATP8 + ATP6	hypothetical protein LOC100203425
*H. magnipapillata*	LOC100214886	1	243	COX2	cytochrome b5 reductase 4
*H. magnipapillata*	LOC100199436	1	283	ψCOX1	toxin-A
*H. magnipapillata*	LOC100211256	1	101	ψCOX1	sox10
*H. magnipapillata*	LOC100210445	1	197	CYTB	dmx-like 1
*H. magnipapillata*	LOC100199718	1	136	ND5	predicted protein (LOC100199718)
*H. magnipapillata*	LOC100209933	1	212	ψCOX1	predicted protein (LOC100209933)
*H. magnipapillata*	LOC100207943	1	132	ψCOX1	predicted protein (LOC100207943)
*H. magnipapillata*	LOC100202784	1	455	ψCOX1	predicted protein (LOC100202784)
*H. magnipapillata*	LOC100199452	2	606	ND4	predicted protein (LOC100199452)
*H. magnipapillata*	LOC100207201	1	105	s-rRNA	viral A-type inclusion protein
*H. magnipapillata*	LOC100208284	1	129	ND1 + ND4	crooked neck-like 1 protein
*H. magnipapillata*	LOC100199804	1	586	tRNA-Met + s-rRNA	spindle assembly 6
*H. magnipapillata*	LOC100210052	1	121	ψCOX1	predicted protein (LOC100210052)
*H. magnipapillata*	LOC100205337	1	317	ND3 + ND4 L	DR1-associated protein 1
*H. magnipapillata*	LOC100201910	1	438	ND4	neuroendocrine convertase 1
*H. magnipapillata*	LOC100211266	1	1120	COX1 + tRNA-Met + s-rRNA	predicted protein (LOC100211266)
*H. magnipapillata*	LOC100210930	1	78	s-rRNA	protein kinase C, delta
*H. magnipapillata*	LOC100208954	1	94	ND3 + ND4 L	predicted protein (LOC100208954)
*H. magnipapillata*	LOC100208526	1	501	ψCOX1 + tRNA-Met + l-rRNA	putative sulphate transporter of the SLC26A11 family
*H. magnipapillata*	LOC100214987	1	610	ATP8 + ATP6	proteasome (prosome, macropain) subunit, beta type, 2
*H. magnipapillata*	LOC100205078	4	2364	CYTB + COX1	MAD homolog 4 interacting transcription coactivator 1
*H. magnipapillata*	LOC100198773	8	6619	COX1 + CYTB + ND4 + ND1 + ND4 L + ND3 + ND6ψCOX1 + tRNA-Met + s-rRNA	mitoferrin-1
*N. vectensis*	XM_001642043	1	136	COX1	predicted protein (NEMVEDRAFT_v1g237784)

Note: NUMTs of *H. magnipapillata* were identified in predicted nuclear genes by searching the positions of NUMTs in seq_gene.md and seq_gene.q files. The NUMT location information of *N. vectensis* was retrieved using the JGI *N. vectensis* v1.0 (Nemve1) Genome Browser (http://genome.jgi-psf.org/Nemve1/Nemve1.home.html).

### The expression of NUMTs suggests its functional relevance

Generally, NUMTs lose their functions and are considered “dead on arrival”. These sequences are likely located in intergenic regions or introns and display no transcriptional activity. However, we observed that NUMTs were co-expressed with adjacent protein-coding genes. In this study, all the *H. magnipapillata* ESTs were retrieved from GenBank to identify NUMT expression. The mitochondrial transcripts were filtered, and authentic ESTs with NUMT insertions were subjected to further analysis. The absence in the *H. magnipapillata* assembly might reflect the use of an individual with a unique NUMT insertion at a specific genomic locus in the cDNA library construction. This unique NUMT insertion event did not occur in the individuals involved in the *Hydra* genome-sequencing project. If the NUMT was expressed through neighbouring promoters, we will be able to identify the corresponding insertion in the EST data. Several of the NUMTs identified in *H. magnipapillata* overlapped with the ESTs. After removing contaminants, we obtained 7 ESTs corresponding to the NUMTs in the partial sequences. BLASTX results show that 4 ESTs (CV151845, CV284212, CX770377, DN243213) partially corresponded to the *COX3* gene and that 1 EST partially corresponded to the *ND6* gene (CA301932). None of these sequences could be perfectly translated due to the presence of mitochondrial stop codons. NUMTs were inserted into their 5’ untranslated regions (UTRs) in 2 other ESTs (CO538443, DN603666). CO538443 is transcribed from a transcript containing the complete exons of *hydramacin-1*, and DN603666 originated from the *arminin 1b* gene.

## Discussion

### Unique mitochondrial genome structure in cnidarians

In our study, the number of NUMTs in the *Hydra magnipapillata* genome was clearly higher than in *Nematostella vectensis* and *Acropora digitifera*. A total of 704 NUMTs were identified in *H. magnipapillata*, representing approximately 0.0470% of the genome (Table 1). The *H. magnipapillata* genome shows a higher cumulative NUMT percentage than the other invertebrate species investigated and a lower NUMT cumulative percentage than that of *Apis mellifera* (0.0861%) [4,5,9].

The structure of cnidarian mtDNA is variable, as both circular and linear mitochondrial molecules have been identified [24,37,38]. *H. magnipapillata* contains two linear mtDNAs, and the *N. vectensis* and *A. digitifera* mtDNAs are single circular molecules. Due to increased susceptibility to exonuclease activity, linear chromosomes are less stable than circular chromosomes and are more easily broken when cells experience damage or the degradation of abnormal mitochondria [23,39]. In addition, in contrast to the two shorter linear DNA chromosomes in *H. magnipapillata*, the circular chromosomes in the other two species must be linearised before integration into the nuclear genome. The frequency of NUMTs might be associated with the number and stability of mitochondrial molecules [4,5]. Therefore, when they enter the nucleus, the mtDNAs in *H. magnipapillata* provide more sources of integrated fragments than those in *N. vectensis* and *A. digitifera*. That is, compared with an animal with a circular mitochondrial chromosome, an animal with a linear mitochondrial chromosome might have more sources to generate mitochondrial fragments that integrate into the nucleus.

**Figure 3:**
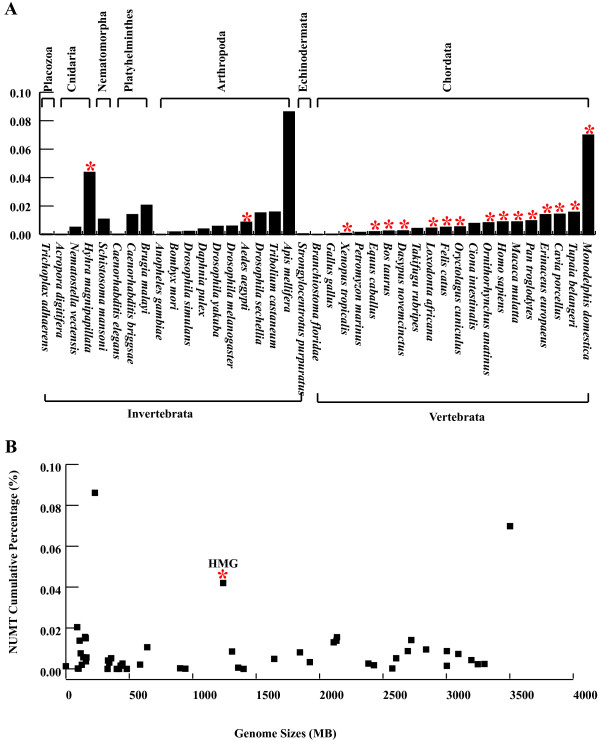
**The ratio of NUMTs to the total size of the nuclear genome (%) in the species concerned and the NUMT cumulative percentage is not correlated to genome sizes. (A)** The ratio of NUMTs to the total size of the nuclear genome (%) in the species concerned. The x-axis contains the name of each species (including species described in the research by Hazkani-Covos [5] and three Cnidaria species), while the y-axis numbers represent the ratio of NUMTs to the total size of the nuclear genome. “*” represents species with large nuclear genomes (lager than 1 Gbp). **(B)** The NUMT cumulative percentage is not correlated with genome size. A log-log scale graph shows the dependence between NUMT cumulative percentage and genome size. Genome size information was retrieved from the review of Einat Hazkani-Covo et al. [5], “*” indicates *H. magnipapillata*.

In the older phyletic lineage of animal including Placozoa, Nematomorpha, and Platyhelminthes, the available data suggest that the ratio of NUMTs to genome size ranges from 0.0002% in *Trichoplax adhaerens* to 0.0204% in *Brugia malayi*[5]. Compared with the species in the older phyletic lineages, *H. magnipapillata* contains a much higher NUMT cumulative percentage. The size of the *H. magnipapillata* genome is of the same order of magnitude as those in the species with the highest NUMT cumulative percentage (Figure 3A). Therefore, the *H. magnipapillata* genome has experienced an exceptionally high frequency of NUMT insertions throughout its evolutionary history. In addition, as shown in Figure 3B, our analysis of 49 animal genomes revealed that no correlation exists between the genome size and NUMT cumulative percentage (Pearson correlation test: *P* = 0.3532). Except in *Monodelphis domestica*, the NUMT cumulative percentage is relatively low in most species with large genomes, particularly in invertebrates. Therefore, *H. magnipapillata* is an exception in that it has a large genome and a high NUMT cumulative percentage.

The evolutionary trend of plant mitochondrial genomes is opposite to that of metazoan mitochondrial genomes, in which the genome has become smaller and more compact [40]. Despite their conserved function in eukaryotes, plant mitochondrial genomes have a variety of unique features compared with those of metazoan animals. In contrast to metazoan animals, which have experienced dramatic reduction of mitochondrial genome size after a prior mitochondria-to-nucleus rate acceleration, the transfer of mitochondrial genomes to the nuclear genomes is still on-going in plants. Because of different transfer rates among plant mitochondrial genomes, their gene repertoires and mitochondrial genome sizes vary considerably. In addition to the ability to transfer DNA fragments to the nucleus, plants also have a propensity to integrate DNA fragments from various foreign sources, including the chloroplast, plastid and nuclear genomes, via intracellular transfer, and from other species via horizontal transfer [41]. Multiple and on-going gene transfer events are responsible for the considerable variations in genome size among plant mitochondrial genomes. Therefore, a comparison of the NUMT content among plants is not feasible due to the complicated evolutionary characteristics of mitochondrial genomes, which are similar for most protist species. Protists have diverged so far from metazoan animals that their mitochondrial genomes display striking diversity in size and complexity and possess large, spacious, gene-rich sequences. The flux from the mitochondria to the nucleus is a widespread and on-going phenomenon in protist species [42]. The mitochondrial genome linearisation in the *Hydra* has split its mitochondrial genome into two linear pieces. The linear mitochondrial genomes in protist species consist of only a single type of DNA molecule, suggesting a distinct mechanism for mitochondrial genome linearisation [43]. Taken together, a comparison of NUMT content among metazoan animals, plants, and protists cannot avoid the bias that results from their distinct mitochondrial genome characteristics.

Considering that the species that have diverged in a short evolutionary time-scale possess similar mitochondrial genome characteristics, we also compared the NUMT content in phylogenetically close species to determine the influences of genome structures on NUMT integration in non-metazoan genomes. To determine whether the variations in the protist NUMT content are influenced by mitochondrial genome structures according to the trend observed in metazoan species, we compared the NUMT content in *Plasmodium* species, which display similar mitochondrial genome characteristics and different mitochondrial genome structures. The genome sequences for six *Plasmodium* species, including four species with linear mitochondrial genomes and two species with circular mitochondrial genomes, have been determined. Our measurements of NUMT content in these species showed that the NUMT content of three species (3 in 4) with linear mitochondrial genomes is more abundant than that of species with circular mitochondrial genomes, suggesting that this trend also follows that observed in metazoan species (Additional file 9: Table S7). *Plasmodium gallinaceum* (GC content: 20.6%; Genome size: 16,913,475 bp; NUMT cumulative percentage: 0.0020%) and *Plasmodium berghei* (GC content: 23.7%, Genome size: 17,952,627 bp; NUMT cumulative percentage: 0%) have similar genome sizes and GC content, but their NUMT cumulative percentage is different, suggesting that the genome size and GC content are not the primary factors to influence the NUMT cumulative percentage.

Another difference between these three species is the genome size. The genome sizes in *N. vectensis* (357 Mbp) and *A. digitifera* (420 Mbp) are of the same order of magnitude, whereas the genome of *H. magnipapillata* (1,240 Mbp) is approximately three times as large as those of the other two species. A comprehensive correlation analysis from multiple genomes shows that the NUMT content is strongly correlated with genome size both in plants and animals [5,44]. Therefore, it is expected that the cumulative size of NUMTs in *H. magnipapillata* will be larger than that of the other two species. However, our results indicate that *H. magnipapillata* is an exception in that it has a large genome size but a high NUMT cumulative percentage. Thus, the NUMT content in *H. magnipapillata* cannot be fully explained by genome size when compared with the NUMT content of other animals. That is, the increased genome size of *H. magnipapillata* is not primarily responsible for the large number of NUMTs. Therefore, our results suggest that linearisation of the mitochondrial genome might primarily reflect the high number of NUMTs in the *H. magnipapillata* genome.

Cnidaria mtDNA displays variations not only in the genome structure but also in genomic components. Introns are frequently identified in cnidarian mitochondrial genomes and have only been reported in cnidarians and sponges among the Metazoa [29,30,45]. Both *N. vectensis* and *A. digitifera* mitochondrial genomes contain an *ND5* intron. The mitochondrial sequences can be integrated in the nuclear genome through two candidate mechanisms: direct DNA transfer and a cDNA intermediate [1,46,47]. Based on examples from plants, prevailing views have focused on cDNA as a vehicle when mitochondrial genes enter the nucleus [47]. Evidence supporting this view includes the finding that integrated nuclear copies of genes that originate from the mitochondrial genome often lack introns. In our study, introns were present in both the *N. vectensis* and *A. digitifera* mitochondrial genomes. The simultaneous presence of both the intron and exons from *ND5* in the NUMT (scaffold_2815 in *N. vectensis*) indicates that direct DNA transfer is one route of mtDNA transfer into the *N. vectensis* genome. Therefore, unlike previously studied genomes exhibiting an absence of introns in the mitochondrial genome, our results provide direct evidence that mtDNA integrates into the nuclear genome through direct DNA transfer.

### NUMT content exhibits dramatic differences in three cnidarian species

An exceptionally high density of NUMTs was detected in the *H. magnipapillata* genome. The number of NUMTs in *H. magnipapillata* clearly exceeded that in *N. vectensis* and *A. digitifera*. All NUMTs exhibited high similarity with their mitochondrial counterparts (92.11 to 100% in *N. vectensis*, 79.34 to 100% in *H. magnipapillata,* and 95.35% in *A. digitifera*). No NUMT duplication events were detected in the *N. vectensis* genome, suggesting that all NUMTs were integrated from the mitochondrial genome. The high sequence similarity between NUMTs and their mitochondrial counterparts indicates that the NUMT integration occurred recently in the *N. vectensis* genome. In *A. digitifera*, only one NUMT was identified, and the scarcity of NUMTs suggests that the *A. digitifera* genome resists NUMT integration. Consequently, although the three species are in the same phylum, their NUMT content and characteristics differ dramatically.

Several early studies suggested that transposable elements or short-dispersed repeats were associated with the on-going integration of mtDNA sequences and their subsequent duplication within the nuclear genome [16-18]. NUMTs are derived from independent insertion events and subsequently undergo further genomic arrangements, resulting in the interruption of older NUMTs through the insertion of repetitive elements. Undoubtedly, duplication increases the NUMT content in *H. magnipapillata*, as evidenced by the absence of NUMT duplications in *A. digitifera* and *N. vectensis*. The transposable element content is much higher in *H. magnipapillata* than in *N. vectensis* and *A. digitifera*[32-34]. Transposable elements have burst three times and are still active in the *H. magnipapillata* genome [33]. Unlike the *H. magnipapillata* genome, transposable elements in *N. vectensi*s comprise a small fractionn of the genome and are all relatively young, and NUMTs are more than 90% similar to their mitochondrial counterparts [32]. In addition, several NUMTs in *H. magnipapillata* were disrupted through transposable elements, while this disruption was not observed in *N. vectensis*. Thus, a low portion of transposable elements is correlated with a low number of NUMTs. In short, these results imply that the variations in the NUMT content in the three cnidarian species might be correlated with the content and activity of transposable elements in their genomes.

### Insertion of NUMTs in nuclear genes and NUMT expression

In the present study, partial sequences in 7 ESTs corresponded to NUMTs. Notably, all NUMT insertions were absent in the original assembly of the *H. magnipapillat*a genome. These differences are polymorphic sites between the genome sequencing and the EST sequencing individuals. The *H. magnipapillata* genome is estimated to contain approximately 0.7% single-nucleotide polymorphisms between alleles, indicating its substantial heterozygosity [33]. It is expected that several polymorphic NUMT insertions might have occurred in different individuals in relatively recent evolutionary times. Therefore, these NUMT insertions might not be fixed in the *H. magnipapillata* genome.

NUMTs are located in regions with low gene content [4,19,36]. This notion has been recently challenged through evidence that the integration of NUMTs in the fish and sponge genomes occurs primarily in known or predicted coding regions [35,48]. In addition, 80% of human-specific NUMTs are integrated into known or predicted genes [21]. In our study, 23 NUMTs were inserted into the introns of *H. magnipapillata*, with 14 NUMTs belonging to known or predicted genes (Table 2). The co-expression of NUMTs with protein-coding genes suggested that NUMT expression might be functionally relevant and important for interactions with neighboring protein-coding genes. Cytochrome b5 reductase 4, toxin A, protein kinase C delta, and proteasome subunit beta type-2 are related to oxidative stress [49-53]; SOX10, proteasome subunit beta type-2, protein kinase C delta, and toxin A are associated with mitochondrial apoptosis or mitochondrial damage [52,54-57]. Mitoferrin-1 functions as an essential importer of iron for the mitochondrial haeme and iron sulphur cluster in erythroblasts and is necessary for erythropoiesis [58]. Six of the 14 known or predicted genes have been associated with mitochondrial activation, suggesting that the proteins that mediate mitochondrial activation have more opportunities to contact the mitochondria. Genes associated with mitochondrial damage are targets for mitochondrial fragment insertion. These results are consistent with previous results suggesting that genetic or environmental factors that increase the frequency of chromosome breaks play a critical role in providing the impetus for the continued invasion of the human genome through mitochondrial DNA [21].

The primary mechanisms responsible for the appearance of new exons are gene duplication, exon shuffling, lateral gene transfer, and alternative splicing [59]. However, the expressed NUMTs detected in the present study suggest that NUMTs are another possible source of new exons. Four ESTs (CV151845, CV284212, CX770377, DN243213) are non-functional because they contain stop codons when translated with both the universal and mitochondrial genetic codes. Although these four transcripts do not encode proteins and their functions are unknown, they correspond to the same gene, COX3. One possible explanation is that NUMTs are transcribed as non-coding RNAs, as previously suggested [60]. Two other ESTs (CO538443, DN603666) contain NUMTs in their 5’UTRs. CO538443 is transcribed from a complete gene, *hydramacin-1*, while DN603666 is only transcribed from part of *arminin 1b*[61,62]. These results suggest that the insertion of NUMT into a gene may have no effect on its transcription and gradually becomes part of the gene, but it might affect the expression of genes encoding proteins. Unlike the previous report that mitochondrial-derived protein-coding exons belong to various functional classes, our results show that NUMT exons belong to the same functional class [63]. Both hydramacin-1 and arminin 1b are novel antimicrobial proteins that were identified during the investigation of the epithelial defence of *H. magnipapillata*[61,62]. Whether all exonised NUMTs in the *H. magnipapillata* genome distribute in immune-related genes requires further investigation. Although only 7 expressed NUMTs were detected in our study, we provide evidence that NUMTs could be expressed in the transcriptome, which might have the potential to affect co-transcriptional genes.

## Conclusions

Our knowledge of mitochondrial DNA integrated into animal nuclear genomes is primarily limited to animals with circular mitochondrial genomes without introns. NUMT studies are not available in animals with linear mitochondrial genomes or those with intron-containing mitochondria. In this study, we provided the first report of NUMT in the phylum Cnidaria, containing mitochondrial genomes with distinct variations in genome structure, including the mtDNA structure (linear or circular) and the presence or absence of introns in protein-coding genes. Our analysis shows that mitochondrial genome linearisation might be responsible for the enrichment of NUMTs in the *Hydra magnipapillata* genome. In addition, co-transposition of exonic and intronic fragments within NUMTs in *Nematostella vectensis* provides direct evidence that mitochondrial sequences might be transposed into the nuclear genome through DNA-mediated fragment transfer. Furthermore, expressed NUMTs might be detected in the transcriptome, suggesting that these sequences might have biological relevance and the potential to affect co-transcriptional genes. Taken together, our results provide valuable information about the impact of different mitochondrial genome structures in NUMT evolution.

## Methods

The mitochondrial genomes were retrieved from the NCBI database for *H. magnipapillata* (NC_011220, NC_011221) and *N. vectensis* (NC_008164) [26,48]. The genome sequences were downloaded from the NCBI database (h7, 28-JAN-2009; ASM20922v1, 22-AUG-2007; Adig_1.0, 28-JUL-2011).

The mitochondrial genome of *A. digitifera* is not available yet. Following the strategies used to study *H. magnipapillata* mtDNAs, we identified the *A. digitifera* mtDNA sequences from the genome assembly [26]. This strategy for assembling the mtDNAs from genome sequencing data was also approved for the mtDNA identification of invertebrates [26,64]. Briefly, we performed homology searches against the *A. digitifera* draft genome using the TBLASTN program with known mitochondrial proteins from closely related species. Subsequently, *A. digitifera* mitochondrial sequences were downloaded from the NCBI database. These sequences were BLASTN searched against the resulting genome sequences obtained from the TBLASTN searches to recover authentic mitochondrial sequences and to assemble mitochondrial genomes. The annotation of *A. digitifera* mtDNA was performed according to the annotation procedure of invertebrate mtDNAs, as described in our previous studies [65,66]. Gene annotation for proteins and rRNAs was performed manually and using the DOGMA program [67]. The tRNA genes were identified using tRNAscan-SE, employing the cove only search mode and the invertebrate mitochondrial genetic code [68].

BLASTN searches of nuclear genome sequences were performed using their corresponding mitochondrial genome sequences, and the maximum expectation value was set to E = 10^-4^, and hits with lengths less than 50 nt were ignored [69]. The total number, size, and locations of NUMTs were determined from the BLASTN results using custom Perl scripts. BLASTN searches were also conducted locally using individual mitochondrial genes as query sequences in each of the *H. magnipapillata*, *N. vectensis*, and *A. digitifera* genome sequences [26,32,34,69]. The sequences of mitochondrial genes were analysed using BioEdit software and custom Perl scripts. Secondary structures of mt-tRNAs and their corresponding NUMT-tRNAs were predicted using the tRNAscan-SE and Mfold programs with default values [70].

If sequences originated from closely spaced regions in the mtDNA but were located away from each other in the nuclear DNA, the fragment between the two NUMTs was extracted from the genome sequence. Because NUMTs are associated with repetitive elements, we used the RepeatMasker program (http://www.repeatmasker.org/) to identify repetitive elements in these extracted fragments. As reported in *Apis mellifera*, NUMTs originating from the same location in mtDNA were considered to be duplicated NUMTs in the nuclear genome [9]. The seq_gene.md and seq_gene.q files containing RefSeq transcript information were downloaded from the NCBI database (http://ftp://ftp.ncbi.nih.gov/genomes/Hydra_magnipapillata/ARCHIVE/BUILD.1.1/mapview/seq_gene.md.gz) and were used to analyse the positions of NUMTs in gene models for *H. magnipapillata*. The presence of predicted genes in a 10-kbp window around each NUMT was considered to determine whether a NUMT was located in a genic or non-genic region (empty, no genes; low gene density, 1–2 genes; high gene density, more than two genes). The location information of NUMTs was retrieved using the JGI *N. vectensis* v1.0 (Nemve1) Genome Browser (http://genome.jgi-psf.org/Nemve1/Nemve1.home.html) and the *A. digitifera* Genome (Ver 1.1) (http://marinegenomics.oist.jp/genomes/downloads?project_id=3).

In total, 164,325 *H. magnipapillata* ESTs were downloaded from GenBank to detect NUMT expression and were searched with mitochondrial genome sequences using BLASTN searches. The BLASTN matches that contained partial sequences not originating from mitochondria were considered to be expressed NUMTs and were further BLASTN searched against the genome sequences to locate their genomic positions.

## Abbreviations

NUMT: Nuclear insertion of mitochondrial sequence; mtDNA: mitochondrial DNA; bp: base pair(s); mt-Chr 1: mitochondrial Chromosome 1; mt-Chr 2: mitochondrial Chromosome 2; EST: Expressed sequence tag; tRNA: transfer RNA; s-rRNA: 12S ribosomal RNA; l-rRNA: 16S ribosomal RNA; ND1-6: and *4 L*, *NADH dehydrogenase subunits 1–6* and *4 L*; Met: Methionine; Trp: Tryptophan; COX 1–3: *Cytochrome c Oxidase subunits 1–3*; UTR: Untranslated region; Mbp: Megabase pairs; CYTB: *Cytochrome b*; NCBI: National center for biotechnology information; ψCOX1: COX1 pseudo-gene.

## Competing interests

The authors declare that there are no competing interests.

## Authors’ contributions

YM and FJ conceived and designed the study. SS, FJ, and YM performed the data analyses and drafted the manuscript. JY and WG assisted in bioinformatic analyses and interpreted the results. All authors have read and approved the manuscript.

## Supplementary Material

Additional file 1: Table S1 and Table S2 NUMTs detected in the *H. magnipapillata* genome, and parameters of alignments returned on BLASTN searches.Start and end indicates the positions of the alignments in the *H. magnipapillata* mitochondrial (mt-Chr 1 and mt-Chr 2) and nuclear genomes. Orientation corresponds to whether integration in the nuclear genome is 5’ > 3’ (+) or 3’ > 5’ (−). The E-value and identity with *H. magnipapillata* mtDNA are indicated as the expected value and % of identity, respectively, for each returned alignment. Click here for file

Additional file 2: Table S3 NUMTs detected in the *N. vectensis* genome, and parameters of alignments returned on BLASTN searches.Start and end indicate the positions of the alignments in the *N. vectensis* mitochondrial (mtDNA) and nuclear genomes. Orientation corresponds to whether integration in the nuclear genome is 5’ > 3’ (+) or 3’ > 5’ (−). The E-value and identity with *N. vectensis* mtDNA are indicated as the expected value and % of identity, respectively, for each returned alignment. Click here for file

Additional file 3: Table S4 NUMTs detected in the *A. digitifera* genome, and parameters of alignments returned on BLASTN searches. Start and end indicate the positions of the alignments in the *A. digitifera* mitochondrial (mtDNA) and nuclear genomes. Orientation corresponds to whether integration in the nuclear genome is 5’ > 3’ (+) or 3’ > 5’ (−). The E-value and identity with *A. digitifera* mtDNA are indicated as the expected value and % of identity, respectively, for each returned alignment. Click here for file

Additional file 4: Table S5 Sequencing and assembly statistics for three cnidarian genomes. Click here for file

Additional file 5: Figure S1 Protein-coding genes in NUMTs could be translated perfectly by mitochondrial and universal codons, and several NUMT tRNAs which could be folded to perfect structures. (**A**) Protein-coding genes (*ND4 L*) in NUMTs could be perfectly translated using mitochondrial and universal codons. (**B**) NUMT tRNAs (e.g., tRNA-Trp) could be folded into perfect structures through simulations using the Mfold web server. Click here for file

Additional file 6: Table S6 A list of NUMT duplications identified in the *H. magnipapillata* genome. Click here for file

Additional file 7: Figure S2 Examples of *H. magnipapillata* NUMTs interrupted by repetitive elements and transposable elements. (**A**) Blue boxes depict NUMTs, and transparent boxes depict repetitive elements. (**B**) Blue boxes depict NUMTs, and transparent boxes depict transposable elements. The numbers (in bold) on top of the blue boxes indicate the beginning and end positions of NUMTs on the scaffold. Click here for file

Additional file 8: Figure S3 Schematic view of predicted genes harbouring NUMTs within introns. (**A**) Schematic view of selected *H. magnipapillata* predicted genes harbouring NUMTs within introns. White boxes depict exons, black boxes depict NUMTs located in the introns, and grey boxes depict UTRs. The white triangle indicates the orientation with respect to the gene. (**B**) Schematic view of the predicted *N. vectensis* genes harbouring NUMTs within introns. Click here for file

Additional file 9: Table S7 NUMT content in protist nuclear genomes. Note: MT size, mitochondrial genome size. NUMT P, NUMT cumulative. Click here for file
