# Supplementary material for: Exceptionally high cumulative percentage of NUMTs originating from linear mitochondrial DNA molecules in the Hydra magnipapillata genome
Source: BMC Genomics. 2013 Jul 4;14:447. doi: 10.1186/1471-2164-14-447 (PMC3716686; doi:10.1186/1471-2164-14-447)
Supplement: Additional file 5: Figure S1 — Protein-coding genes in NUMTs could be translated perfectly by mitochondrial and universal codons, and several NUMT tRNAs which could be folded to perfect structures. (A) Protein-coding genes (ND4 L) in NUMTs could be perfectly translated using mitochondrial and universal codons. (B) NUMT tRNAs (e.g., tRNA-Trp) could be folded into perfect structures through simulations using the Mfold web server. [file 1471-2164-14-447-S5.pdf]

Figure S1

A

>NW_002194340.1_ND4L

	ATGTTATTTGACTTTAATTTATTAGTAGTAATTATGTTTTGTATTAGTATTATAGGTATAATCATAAATAGAAGTAATATAATATTAATTTTAGTTAGTATA
standard codes	M L F D F N L L V V I M F C I S I I G I I I N R S N I I L I L V S I
mitochondrial code	M L F D F N L L V V I M F C I S I I G I I I N R S N I I L I L V S I
	GAAATTTTATTACTATCAATTTCAATGAACTTTATGTTAACCTCATTGACTAATTTTTCTTTAGAGGGACAAATAATAGCAATATATGTAATAACTATAGCT
	E I L L L S I S M N F M L T S L T N F S L E G Q I I A I Y V I T I A
	E I L L L S I S M N F M L T S L T N F S L E G Q I I A I Y V I T I A
	GCTATTGAATCAGCTATTGGATTATCAATAATAGTAGCTTTTTATAAAATAAAAGGATCTATTTCTTTAAAATTATTAAATTTATTAAAAGGATAA
	A I E S A I G L S I I V A F Y K I K G S I S L K L L N L L K G *
	A I E S A I G L S I I V A F Y K I K G S I S L K L L N L L K G *

Yo

B

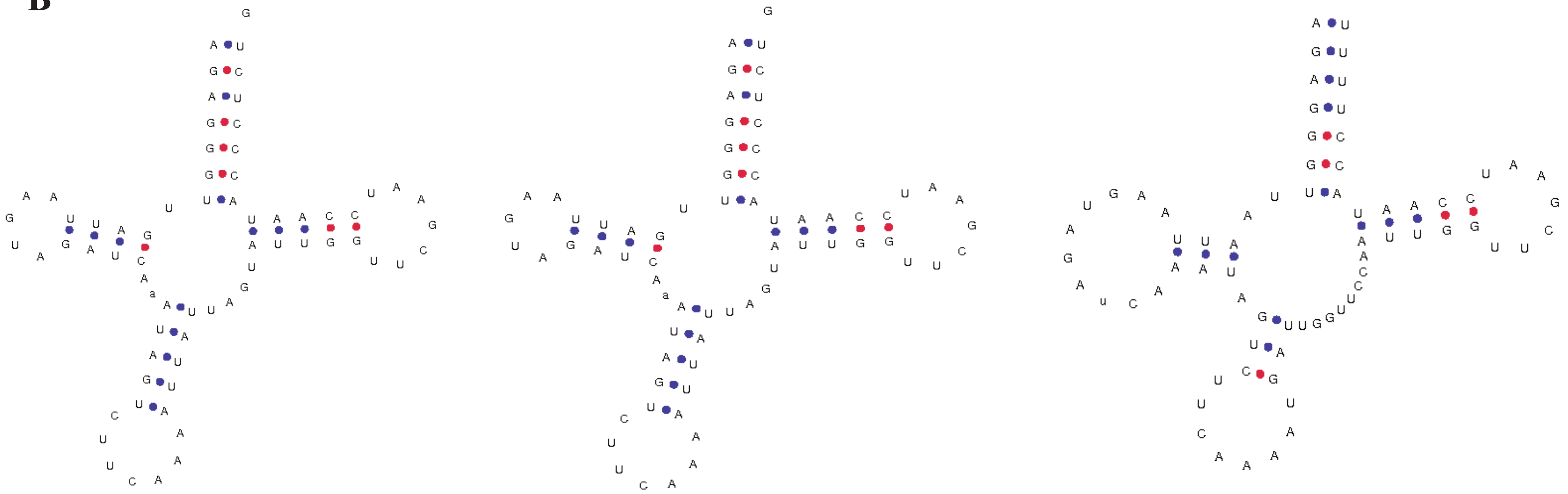

NC_011220-Trp-tRNA | **NW_002143484.1-Trp-tRNA** | **NW_002158220.1-Trp-tRNA**
