# Supplementary figures and images for: Exceptionally high cumulative percentage of NUMTs originating from linear mitochondrial DNA molecules in the Hydra magnipapillata genome
Source: BMC Genomics. 2013 Jul 4;14:447. doi: 10.1186/1471-2164-14-447 (PMC3716686; doi:10.1186/1471-2164-14-447)

Figure S2

A

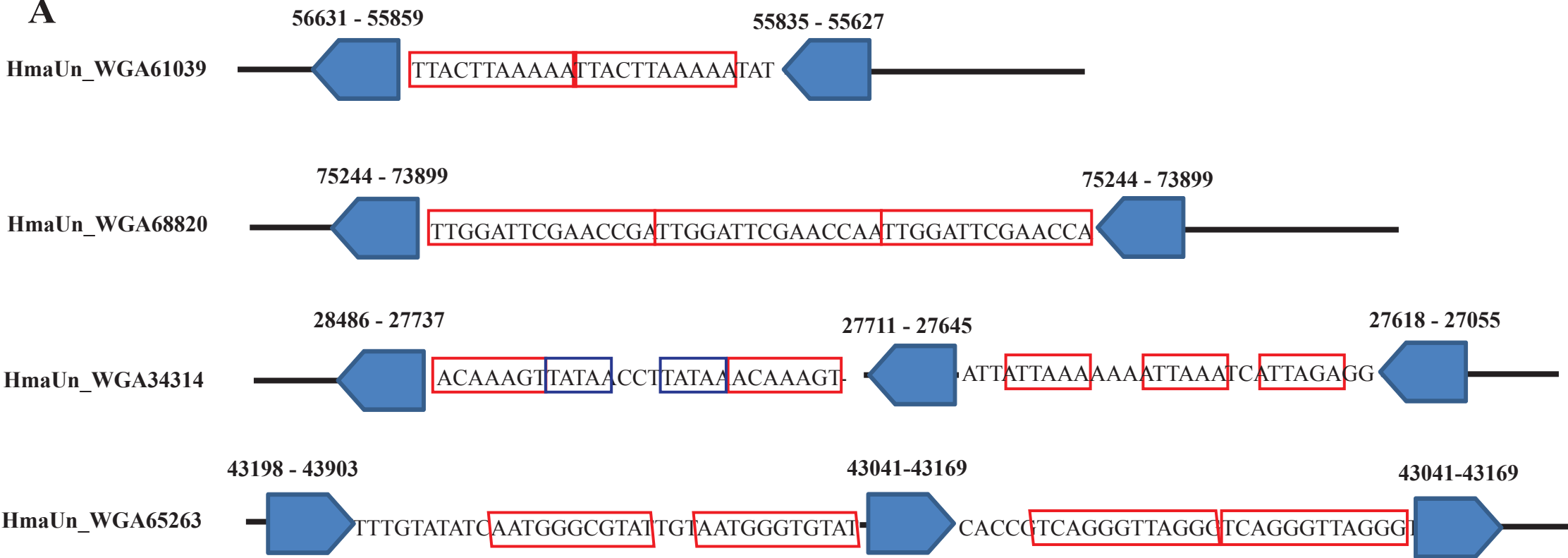

B

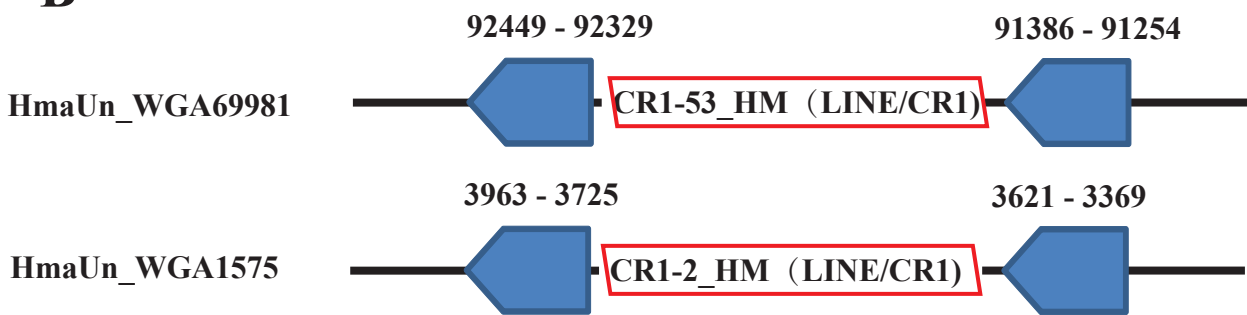

Supplement: Additional file 7: Figure S2 — Examples of H. magnipapillata NUMTs interrupted by repetitive elements and transposable elements. (A) Blue boxes depict NUMTs, and transparent boxes depict repetitive elements. (B) Blue boxes depict NUMTs, and transparent boxes depict transposable elements. The numbers (in bold) on top of the blue boxes indicate the beginning and end positions of NUMTs on the scaffold. [file 1471-2164-14-447-S7.pdf]
