# Supplementary material for: Exceptionally high cumulative percentage of NUMTs originating from linear mitochondrial DNA molecules in the Hydra magnipapillata genome
Source: BMC Genomics. 2013 Jul 4;14:447. doi: 10.1186/1471-2164-14-447 (PMC3716686; doi:10.1186/1471-2164-14-447)
Supplement: Additional file 8: Figure S3 — Schematic view of predicted genes harbouring NUMTs within introns. (A) Schematic view of selected H. magnipapillata predicted genes harbouring NUMTs within introns. White boxes depict exons, black boxes depict NUMTs located in the introns, and grey boxes depict UTRs. The white triangle indicates the orientation with respect to the gene. (B) Schematic view of the predicted N. vectensis genes harbouring NUMTs within introns. [file 1471-2164-14-447-S8.pdf]

Figure S3

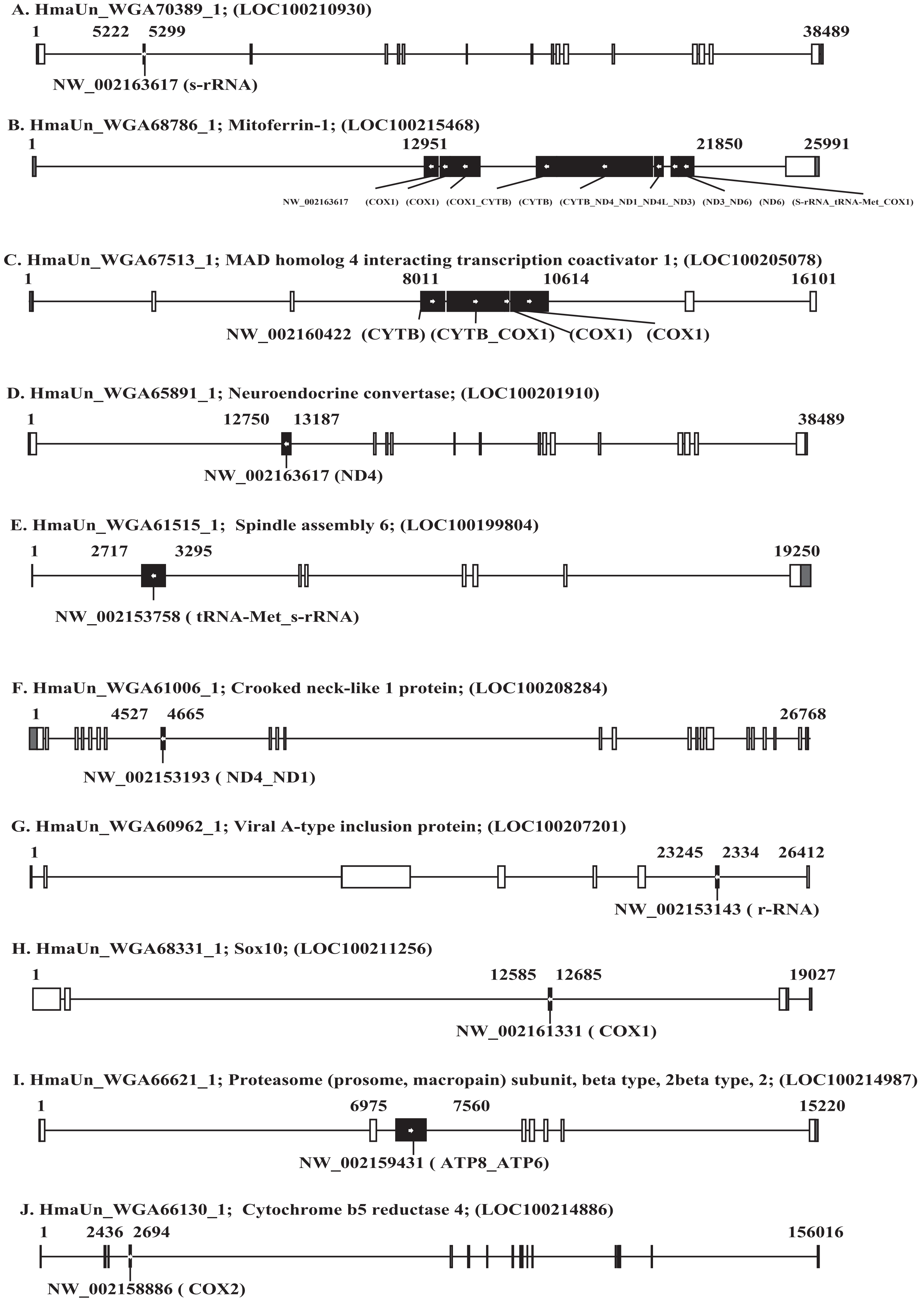

A. HmaUn_WGA70389_1; (LOC100210930)
1
5222
5299
38489
NW_002163617 (s-rRNA)
B. HmaUn_WGA68786_1; Mitoferrin-1; (LOC100215468)
1
12951
21850
25991
NW_002163617
(COX1)
(COX1)
(COX1_CYTB)
(CYTB)
(CYTB_ND4_ND1_ND4L_ND3)
(ND3_ND6)
(ND6)
(S-rRNA_tRNA-Met_COX1)
C. HmaUn_WGA67513_1; MAD homolog 4 interacting transcription coactivator 1; (LOC100205078)
1
8011
10614
16101
NW_002160422
(CYTB)
(CYTB_COX1)
(COX1)
(COX1)
D. HmaUn_WGA65891_1; Neuroendocrine convertase; (LOC100201910)
1
12750
13187
38489
NW_002163617 (ND4)
E. HmaUn_WGA61515_1; Spindle assembly 6; (LOC100199804)
1
2717
3295
19250
NW_002153758 (tRNA-Met_s-rRNA)
F. HmaUn_WGA61006_1; Crooked neck-like 1 protein; (LOC100208284)
1
4527
4665
26768
NW_002153193 (ND4_ND1)
G. HmaUn_WGA60962_1; Viral A-type inclusion protein; (LOC100207201)
1
23245
2334
26412
NW_002153143 (r-RNA)
H. HmaUn_WGA68331_1; Sox10; (LOC100211256)
1
12585
12685
19027
NW_002161331 (COX1)
I. HmaUn_WGA66621_1; Proteasome (prosome, macropain) subunit, beta type, 2beta type, 2; (LOC100214987)
1
6975
7560
15220
NW_002159431 (ATP8_ATP6)
J. HmaUn_WGA66130_1; Cytochrome b5 reductase 4; (LOC100214886)
1
2436
2694
156016
NW_002158886 (COX2)
